# Effect of phone call distraction on the performance of medical students in an OSCE

**DOI:** 10.1186/s12909-022-03215-y

**Published:** 2022-04-20

**Authors:** Justus F. Toader, Robert Kleinert, Thomas Dratsch, Louisa Fettweis, Nadja Jakovljevic, Martina Graupner, Moritz Zeeh, Anna C. Kroll, Hans F. Fuchs, Roger Wahba, Patrick Plum, Christiane J. Bruns, Rabi R. Datta

**Affiliations:** 1 Department of General, Visceral, Cancer And Transplant Surgery, University of Cologne, Cologne, Germany; 2 Department of General and Visceral Surgery, Evangelisches Klinikum Bethel, Universitätsklinikum OWL, Bielefeld, Germany

**Keywords:** OSCE, Medical education, Distraction

## Abstract

**Background:**

The usage of smartphones in the daily clinical routine is an essential aspect however it seems that they also present an important distractor that needs to be evaluated. The aim of this prospective study was the evaluation of the influence of phone calls as distractors on the performance levels of medical students during an objective structured clinical examination (OSCE), simulating the normal clinical practice.

**Methods:**

As the goal of an OSCE presents the examination of clinical skills of medical students in a realistic setting, more than 100 students recruited from the university hospital of Cologne participated in either OSCE I or II. During the OSCE I intravenous cannulation was simulated while OSCE II simulated an acute abdominal pain station. Participants had to perform each of these stations under two circumstances: a normal simulated OSCE and an OSCE station with phone call distraction. Their performance during both simulations was then evaluated.

**Results:**

In OSCE I students achieved significantly more points in the intravenous cannulation station if they were not distracted by phone calls (M=6.44 vs M=5.95). In OSCE II students achieved significantly more points in the acute abdominal pain station if they were not distracted by phone calls (M=7.59 vs M=6.84). While comparing only those students that completed both stations in OSCE I/II participating students achieved significantly more points in both OSCE I and II if they were not distracted by phone calls.

**Conclusion:**

The presented data shows that phone call distraction decreases the performance level of medical students during an OSCE station. Therefore, it is an indicator that distraction especially for younger doctors should be held to a minimum. On a second note distraction should be integrated in the medical education system as it plays an important role in clinical routine.

## Background

In the present day, the usage of smartphones and similar devices during medical work makes up a large part of the average clinical daily routine [1]. It seems to be clear that there is a perpetual need for availability to others in a hospital setting. Smartphones have the ability to provide resources and means of communication for medical professionals however suspicions have been raised on its potential to cause distractions and disruptions on one’s performance. This specifically leads to speculation over the impact smartphones have in the daily clinical routine. To what extent do smartphones interfere with cognitive capacity, concentration and even clinical performance?

Multiple studies have discussed the influence and effects of cell phones during different tasks, for example driving, where in those using phones, the driver performance showed to be significantly decreased and the distraction increased [2]. The mere presence of one’s own smartphone was shown to adversely affect cognitive capacity [3]. A study evaluating the distraction of phone calls during a laparoscopic surgery performed by novice surgeons was published in 2017. It showed that phone calls during laparoscopic surgery have a negative effect on the performance of the novice surgeons, which made significantly more surgical and cognitive errors [4]. It is of importance to investigate whether phone calls during clinical routine distract doctors, especially those of inexperience and possibly cause them to produce more mistakes.

Consequently, the question arises whether the unrestricted accessibility by telephone affects medical staff in the daily work. Testing of such a correlation is difficult due to ethical restrictions. Hence we conducted an experiment in a predefined and valid environment: Objective structured clinical examinations (OSCEs) have shown to be valid and reliable examination methods for medical students that evaluate clinical tasks and simulate daily practice [5]. An OSCE is a realistic simulation of a daily situation doctors encounter during medical practice, such as inserting an intravenous cannula or performing a general examination. It earned its value as a means to evaluate the practical abilities and skills of medical students [5, 6].

The aim of this study is to evaluate the influence of phone calls as distractors on the performance levels of medical students during an OSCE, simulating the normal clinical practice.

## Material and Methods

### Ethics and Data management

Ethics Committee approval was obtained before the study (Ethics Committee, University of Cologne) and the current study adheres to the criteria of our local ethics committee (No. 19-1327). Written informed consent was given by all participants before study inclusion. All data were anonymized before analyses and stored according to the data management requirements by our local Ethics Committee.

### Participants OSCE I and OSCE II

All students were recruited at the University Hospital of Cologne. One hundred and sixty-one medical students (76 male, 85 female; mean age = 23.7, age range: 20–33) participated in the study as part of their OSCE I examination.

One hundred and forty-seven medical students (61 male, 86 female; mean age = 25.7; age range: 22–43) participated in the study as part of their OSCE II examination.

### General Description of OSCE

The goal of an OSCE is to examine the clinical skills of medical students in a realistic setting. It takes part in semester 1 and 5. In OSCE I, students completed a total of 8 stations. In OSCE II, students completed a total of 14 stations. The general procedure was the same in both OSCEs. At the beginning of the OSCE, students were all instructed to wait in front of one of 8 (OSCE I) or 14 (OSCE II) rooms. Pasted to the door of each room was a short description of the patient in each room and the task to be performed. Then, an alarm would ring to mark the start of the OSCE. Students then had one minute to read the instructions pasted to the door. After one minute, a second alarm would ring to signal students to enter the room. Students then had five minutes to complete the task in the room. After five minutes had passed, another alarm signalled students that they now had one minute to leave the room and read the instructions in front of the next room.

### Design and Materials OSCE I

In the OSCE I examination, the effect of phone call distraction on the performance in the intravenous cannulation station was analysed. In the intravenous cannulation station, students had to first assemble all the materials needed for an intravenous cannulation, perform the cannulation, and then start an intravenous therapy. Table 1 lists all the correct steps students needed to perform and the amount of points awarded for each step. A total of 10 points could be earned if all steps were performed correctly. Partial points were also awarded.

**Table 1:** Correct steps to be performed in the intravenous cannulation station and the points awarded for each step

Step	Correct	Partial
Assembling Materials		
Peripheral venous cathether	0.16	
Tourniquet	0.16	
Alcohol	0.16	
Band-aid	0.16	
Swab	0.16	
Sharps container	0.20	
Putting on gloves (partial if hand disinfection missing)	1.0	0.5
Putting on the tourniquet (partial if kept on for too long)	0.5	0.25
Taking off the tourniquet (partial if taken off after needle has been removed)	0.5	0.25
Cleaning the patient’s skin with alcohol	0.5	
Letting the alcohol dry for 30 seconds	0.5	
Inserting the needle (partial if only successful on second try)	0.5	0.25
Sterile insertion of the needle	0.5	0.25
Disposing the needle into the sharps container (partial if needle is placed on a tray)	1.0	0.5
Fixing the catheter in place	1.0	
Starting the intravenous therapy	1.0	
Informing the patient about the procedure	0.5	
Performing all steps in a structured manner	1.0	
Sanitizing the hands after the procedure has been completed	0.5	

To investigate the effect of phone call distraction, the intravenous cannulation station was completed twice: once with and once without phone call distraction. The order of the two stations was randomized. Thus, 50.0% of students first completed the station with phone call distraction and 50.0% of students first completed the station without phone call distraction.

In the regular OSCE station, students performed the task without interruption. In the phone call distraction condition, students were interrupted twice by a phone call. In the regular OSCE station, students had one minute to read the instructions pasted to the door and five minutes to complete the task. To account for the time lost due to the two phone calls, students in the phone call distraction condition were instructed to immediately enter the room after they had finished reading the instructions and to not wait for the second alarm. Because most students took only around thirty seconds to read the instructions, this left students with around five minutes and thirty seconds to complete the task in the phone call distraction condition.

Upon entering the room, students were handed a mobile phone and students then started to assemble the materials for the intravenous cannulation. Thirty seconds after the second alarm, the phone rang for the first time. On the other side was an actor playing a nurse: “Here is nurse X. Patient Mr./Ms. X, who is supposed to undergo surgery tomorrow, has a blood glucose level of X. He/she is feeling well. I am just calling to tell you that I will administer insulin according to standard protocol.” If students had further questions, they were assured that this was standard protocol and the nurse quickly ended the conversation. Students then continued to perform the intravenous cannulation. Three different names were used for the patient (Fischer, Weber, or Mutlu). The blood glucose level was also slightly varied for each call.

Two minutes after the second alarm, the phone rang for the second time. This time, an actor playing an attending was on the other side: “Here is attending Mr./Ms. X. Anesthesiology wants to know the blood glucose level of the patient who is due for surgery tomorrow. What is the name of the patient and what is the blood glucose level?” If the student was unable to answer the questions, the attending told the student that he/she was going to call someone else to get the information. Students then continued to complete the OSCE station without interruption.

### Design and Materials OSCE II

In the OSCE II examination, the effect of phone call distraction on the performance in the acute abdominal pain station was analysed. In the acute abdominal pain station, students had to take the medical history of a patient with acute abdominal pain, perform a physical exam, and make a correct diagnosis. Three different diseases were portrayed by the actors: acute cholecystitis, acute diverticulitis, or acute appendicitis.

Table 2 lists all the correct steps students needed to perform and the amount of points awarded for each step. Again, a total of 10 points could be earned if all steps were performed correctly. Partial points were also awarded.

**Table 2:** Correct steps to perform in the acute abdominal pain station and the points awarded for each step

Step	Correct	Partial
Medical History		
Pain (where, how, when, why)	1.0	0.5
Vegetative Symptoms (nausea, vomiting, fever, bowel movements)	1.0	0.5
Prior medical history (prior illnesses, prior operations)	1.0	0.5
Medications and allergies	1.0	0.5
Physical Examination		
Correct positioning of the patient	0.8	
Inspection (verbalizing the results)	0.8	
Auscultation of the abdomen in all four quadrants	0.8	
Percussion of the abdomen	0.8	
Palpation of the abdomen (starting in a non-painful region)	0.4	
Signs (depending on the scenario)	0.4	
Diverticulitis: rebound tenderness		
Cholecystitis: Murphy’s sign		
Appendicitis: Blumberg’s sign		
Diagnosis		
Naming the correct diagnosis and further diagnostic steps	1.0	0.5
Communication		
Communication with the patient (professional behavior, clear communication, showing empathy, hygiene)	1.0	0.5

To investigate the effect of phone call distraction, the acute abdominal pain station was completed twice: once with and once without phone call distraction. The order of the two stations was randomized. Thus, 43.5% of students first completed the station with phone call distraction and 56.5% of students first completed the station without phone call distraction. To ensure that students never completed the same scenario twice in a row, a different scenario was chosen for the second time a student completed the station. For example, if a student had to examine a patient with acute cholecystitis in the phone call distraction condition, the same student had to examine a patient with acute appendicitis in the regular OSCE station without distraction.

In the regular OSCE station, students performed the task without interruption. In the phone call distraction condition, students were interrupted twice by a phone call. The content of the phone calls was the same as in the OSCE I.

Upon entering the room, students were handed a mobile phone and students then started to talk to the patient. Ninety seconds after the second alarm, the phone rang for the first time. In the first call, a nurse informed students about the name and blood glucose levels of a patient and asked whether it was okay to administer insulin. Three different names were used for the patient (Fischer, Weber, or Mutlu). The blood glucose level was also slightly varied for each call.

After the call, students proceeded to talk to the patient. Three minutes after the second alarm, the phone rang for the second time. In the second call, an attending asked the students for the name and blood glucose levels of the patient. Students could then complete the OSCE station without interruption.

### Procedure OSCE I and OSCE II

One week before their OSCE examination, students attended a lecture explaining the details of this year’s OSCE examination. At the lecture, students were also informed about this study. At the end of the lecture, students gave written informed consent to participate in the study and completed a questionnaire with several demographic questions. Because only 8 (OSCE I) or 14 (OSCE II) students could participate in one session at the same time, both OSCE I and OSCE II were spread out over multiple sessions on several days. Students were assigned to their sessions by the university and arrived for their sessions one hour earlier to check-in. After a short introduction, students then put on their white coats and waited for their session to begin. Students then completed all the OSCE stations in their session, which took around 60 minutes (OSCE I) or 90 minutes (OSCE II), respectively. The OSCE stations of this study were mixed in between the regular OSCE stations and were arranged so that students never completed the same station twice in a row. In the OSCE I, the station with phone call distraction and the regular OSCE station were separated by three other OSCE stations. In the OSCE II, the OSCE station with phone call distraction and the regular OSCE station were separated by either five or seven other OSCE stations. After students had completed all OSCE stations, a pre-taped applause signalled the end of the OSCE. Students who had successfully completed OSCE I received a certificate with detailed feedback about their OSCE performance immediately after OSCE I. Students who had successfully completed OSCE II received an email with detailed feedback several days after the OSCE.

### Scoring

Based on the checklists in Tables 1 and 2, the performance of the students was rated by examiners in the room. In addition to the checklist, the examiners also recorded whether the students correctly remembered the name of the patient in the second phone call and whether they could correctly reproduce the patient’s blood glucose level. Also, examiners noted whether students completed the station in the given time limit.

### Actors and Mannequin

In the OSCE I, students performed the intravenous cannulation on a mannequin. In the OSCE II, the three diseases (acute appendicitis, acute cholecystitis, and acute diverticulitis) were portrayed by professional actors who had been specifically coached in portraying the diseases for the OSCE examination.

### Statistical analysis

A statistical power analysis was performed for sample size estimation for the randomized controlled experiment with the medical students. With an alpha = .05 and power = .80, the projected sample size needed to detect a medium effect (Cohen’s d of 0.5) for the within-group comparisons was N = 34 (GPower 3.1). Data were analysed using the Statistical Package for the Social Sciences (SPSS, Version 25; IBM, 2017). Comparisons were conducted using *t* tests.

## Results

### Effect of Phone Call Distraction

To test whether medical students performed worse in the OSCE when they were distracted by the phone call, we compared the points achieved in the regular OSCE station with the points achieved in the OSCE station with phone call distraction using paired *t* tests.

In the OSCE I, there was a significant difference in the number of points between the regular OSCE station (*M* = 6.44, *SD* = 1.77) and the OSCE station with phone call distraction (*M* = 5.95, *SD* = 1.69), *t*(160) = 2.96, *p* = .004, *d* = 0.23, indicating that students achieved more points in the intravenous cannulation station if they were not distracted by the phone calls. Table 3 shows the differences between both groups for all individual items on the OSCE checklist. As the results show, students in the phone call distraction condition scored significantly less points with regard to assembling materials, putting on gloves and the tourniquet, and inserting the needle.

**Table 3:** Differences in the number of points achieved in the OSCE station with and without phone call distraction. Negative values indicate less points in the OSCE station with phone call distraction.

Step	Mean Difference	*p*
Assembling Materials		
Peripheral venous cathether	-0.00199	.158
Tourniquet	-0.01292	**.001**
Alcohol	-0.01391	**<.001**
Band-aid	0.00099	.797
Swab	-0.01491	**.002**
Sharps container	-0.0248	**.002**
Putting on gloves (partial if hand disinfection missing)	-0.0776	**.01**
Putting on the tourniquet (partial if kept on for too long)	-0.0295	**.016**
Taking off the tourniquet (partial if taken off after needle has been removed)	-0.02019	.366
Cleaning the patient’s skin with alcohol	-0.0124	.451
Letting the alcohol dry for 30 seconds	-0.0186	.181
Inserting the needle (partial if only successful on second try)	-0.06832	.**001**
Sterile insertion of the needle	-0.0155	.494
Disposing the needle into the sharps container (partial if needle is placed on a tray)	-0.0683	.144
Fixing the catheter in place	-0.0373	.436
Starting the intravenous therapy	0.0031	.942
Informing the patient about the procedure	-0.0311	.114
Performing all steps in a structured manner	-0.0342	.26
Sanitizing the hands after the procedure has been completed	-0.0124	.451

In the OSCE II, there was also a significant difference in the number of points between the regular OSCE station (*M* = 7.59, *SD* = 1.20) and the OSCE station with phone call distraction (*M* = 6.84, *SD* = 1.23), *t*(146) = 6.55, *p* < .001, *d* = .55, indicating that students achieved more points in the acute abdominal pain station if they were not distracted by the phone calls. Table 4 shows the differences between both groups for all individual items on the OSCE checklist. As the results show, students in the phone call distraction condition scored significantly less points with regard to taking the prior medical history (including medications and allergies), inspection, auscultation, percussion. Only with regard to correct positioning did students in the phone call distraction condition score more points.

**Table 4:** Differences in the number of points achieved in the OSCE station with and without phone call distraction. Negative values indicate less points in the OSCE station with phone call distraction.

Step	Mean Difference	*p*
Medical History		
Pain (where, how, when, why)	0.0136	.573
Vegetative Symptoms (nausea, vomiting, fever, bowel movements)	-0.0408	.287
Prior medical history (prior illnesses, prior operations)	-0.1463	**<.001**
Medications and allergies	-0.1531	**<.001**
Physical Examination		
Correct positioning of the patient	0.098	**<.001**
Inspection (verbalizing the results)	-0.1469	**<.001**
Auscultation of the abdomen in all four quadrants	-0.0435	**.032**
Percussion of the abdomen	-0.185	**<.001**
Palpation of the abdomen (starting in a non-painful region)	-0.0027	.707
Signs (depending on the scenario)	-0.0218	.195
Diverticulitis: rebound tenderness		
Cholecystitis: Murphy’s sign		
Appendicitis: Blumberg’s sign		
Diagnosis		
Naming the correct diagnosis and further diagnostic steps	-0.0476	.136
Communication		
Communication with the patient (professional behavior, clear communication, showing empathy, hygiene)	-0.0068	.495

To account for the fact that the phone calls took away time from the students to complete the OSCE station, students were given more time to complete the OSCE station in the phone call distraction condition. However, to ensure that the difference in points between the phone call distraction condition and the regular condition was not due to the fact that students were not able to finish the OSCE station in the given time and therefore achieved less points, we compared the number of students who were not able to finish the OSCE stations between both conditions. In the OSCE I, 75.8% of students (122 of 161) did not finish the regular OSCE station and 75.8% of students (122 of 161) did not finish the OSCE station with phone call distraction. In the OSCE II, 14.3% of students (21 of 147) did not finish the regular OSCE station and 23.1% of students (34 of 147) did not finish the OSCE station with phone call distraction.

In the OSCE I, analysing only the students who completed both stations (n = 13), there was a significant difference in the number of points between the regular OSCE station (*M* = 8.48, *SD* = 0.89) and the OSCE station with phone call distraction (*M* = 7.39, *SD* = 0.91), *t*(12) = 3.84, *p* = .002, *d* = 1.07, indicating that students achieved more points in the intravenous cannulation station if they were not distracted by the phone calls.

In the OSCE II, analysing only the students who completed both stations (n = 101), there was a significant difference in the number of points between the regular OSCE station (*M* = 7.61, *SD* = 1.16) and the OSCE station with phone call distraction (*M* = 6.92, *SD* = 1.23), *t*(100) = 5.21, *p* < .001, *d* = 0.53, indicating that students achieved more points in the acute abdominal pain station if they were not distracted by the phone calls. We therefore conclude that the difference between the regular OSCE stations and the OSCE stations with phone call distraction was not due to the fact that students were not able to finish the OSCE station in the phone call distraction condition.

In the OSCE II, students gathered the medical history of the patient and performed a physical exam. There was a significant difference in the number of points for taking of the medical history between the regular OSCE station (*M* = 2.77, *SD* = 0.76) and the OSCE station with phone call distraction (*M* = 2.44, *SD* = 0.80), *t*(146) = 4.22, *p* < .001, *d* = 0.36, indicating that students achieved more points in the acute abdominal pain station if they were not distracted by the phone calls.

There was also a significant difference in the number of points for performing the physical exam between the regular OSCE station (*M* = 3.12, *SD* = 0.68) and the OSCE station with phone call distraction (*M* = 2.82, *SD* = 0.70), *t*(146) = 4.82, *p* < .001, *d* = 0.40, indicating that students achieved more points in the acute abdominal pain station if they were not distracted by the phone calls.

### Correlation between performance in the regular OSCE and OSCE with Phone Call Distraction

To analyze whether performance in the regular OSCE station was related to the performance in the OSCE station with phone call distraction, we correlated the points in the regular OSCE station with the points in the OSCE station with phone call distraction. In the OSCE I, there was a significant correlation between the points in the regular OSCE station and the OSCE station with phone call distraction, *r*(161) = .262, *p* = .001.

In the OSCE II, there was also a significant correlation between the points in the regular OSCE station and the OSCE station with phone call distraction, *r*(147) = .348, *p* < .001. This indicates that in both OSCE I and OSCE II students who achieved more points in the regular OSCE station also achieved more points in the OSCE station with phone call distraction.

### Recall of Information from the Phone Call

In both OSCE I and OSCE II, students were asked whether they could recall the patient’s name and blood glucose level. Table 5 shows the recall rate for OSCE I and OSCE II.

**Table 5:** Recall rate for the information from the phone call for OSCE I and OSCE II

	Patient’s Name	Blood Glucose Level
OSCE I	28.0% (45 of 161)	46.0% (74 of 161)
OSCE II	32.7% (48 of 147)	57.1% (84 of 147)

## Discussion

**Fig. 1:**
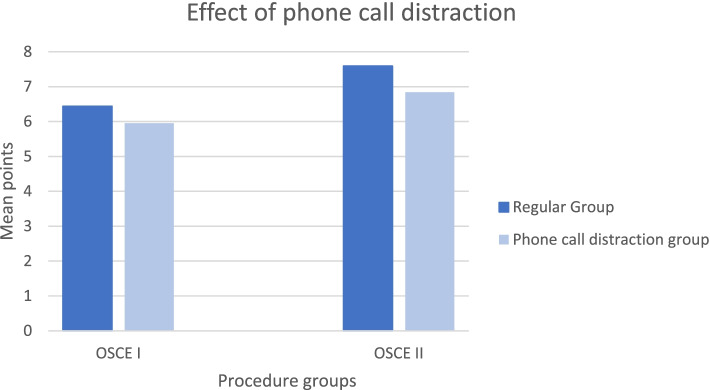
The effects of phone call distractions on students completing OSCE-stations

This study evaluated the influence of phone calls as distractors on the performance of medical students during an OSCE, which can be validated as a simulation of clinical practice. The collected data showed that students did significantly worse when being called while performing either intravenous cannulation in comparison with the regular group. Similar results were found between the two groups for OSCE II regarding the acute abdominal pain station (Fig. 1).

This is consistent with widespread research analysing distraction in the medical field. Several studies have investigated the impact of distraction in different scientific areas and shown a significant increase of errors and mistakes associated with increased levels of distraction [7, 8]. A recent study by Yang et al. has shown that under certain circumstances phone calls during laparoscopic surgery lead to a decreased performance level and increased stress levels [4].

**Fig. 2:**
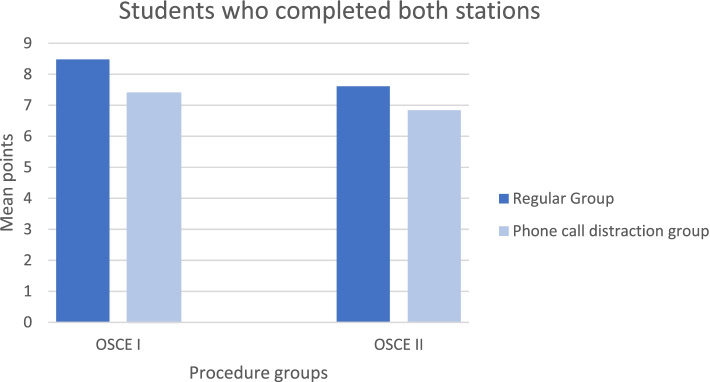
The effects of phone call distractions on students which completed OSCE- stations

This study also showed that a significant difference in points was achieved in comparing the phone call distraction group with the normal group of those that were able to finish the OSCE station. The distraction group scored significantly less points compared to the normal group. This was done to ensure that the difference in points was not only accounted for by not finishing the OSCE station  (Fig. 2).

Since an OSCE station is meant to be evaluating practical clinical abilities while simulating daily medical tasks it was used as a portrayal of the clinical setting. The environment in which doctors work on a daily setting houses several distractors which are led especially by auditory stimuli. Studies evaluating auditory stimuli in the surgical field have shown that an auditory stimulus has greater impact on performance than visual stimuli [9].

Studies analysing tasks that require a higher level of concentration and focus like laparoscopic surgery or endoscopy have shown the negative influence of distraction [10–12]. In comparison our study doesn’t evaluate specialized tasks but more standard routine medical procedures like anamnesis, where distraction should also be seen as one major influencing important factor. When called, doctors are expected to engage, answer or decide about patient related issues which makes phone calls not only intermittent auditory but also cognitive distractors. As a result, it comes to an attention shift from the current task to the new intruding task, forgetting essential parts of the primary task being performed, which results in errors [7, 13].

Another question becoming evident from the results is the factor of educating and teaching medical students about distraction in the medical field. Srisarajivakul et al. tried to integrate the approach of disruptive behaviour into an OSCE station which can also be seen as a distracting agent [14]. Analysing the points achieved in the different domains of the checklist of the two OSCEs revealed that the students scored significantly less points in essential steps during the venous canulation and the abdominal pain station. Steps like inserting the needle correctly, taking a proper medical history, correct auscultation and percussion as well as inspection have a major influence on the clinical performance. Further studies should be done implementing distractions in medical education and thereby preparing medical students for clinical daily routine.

Even though there will always be distractions because the interrupting agent has particular aim that has to be answered, interruptions and distractions should be kept to a minimum as it is shown to lead to a significant increase of errors. That being said the need for more research of how to handle distractions in the medical field has to be done, especially for young unexperienced doctors [7, 15].

### Limitations

The limitation of this study involves the OSCE and in how far it is an adequate representation of the normal clinical practice. It can be seen as a tool to teach and validate medical abilities that are necessary to work as a medical doctor, but it will always just be a simulation and therefore not fully display a real-life situation.

Even though in OSCE II the tasks of the abdominal pain station have been swapped to counteract a proposed training effect performing the same stations twice, this can still be seen as a limitation of this study design.

## Conclusion

Phone call distraction decreases the performance level of medical students during an OSCE station. On the one hand phone calls during medical work should be held to a minimum and on the other hand distraction should be integrated in the medical education as it is a common factor that especially novice doctors will encounter.

## Data Availability

The datasets used and/or analysed during the current study are available from the corresponding author on reasonable request.

## Abbreviation

OSCE: Objective structured clinical examination

## References

[1] Dittrich F, Back DA, Harren AK, et al. Smartphone and App Usage in Orthopedics and Trauma Surgery: Survey Study of Physicians Regarding Acceptance, Risks, and Future Prospects in Germany. JMIR Form Res. 2020;4(11). 10.2196/14787 10.2196/14787 PMC7735902 33252340

[2] Caird JK, Willness CR, Steel P, Scialfa C (2008). A meta-analysis of the effects of cell phones on driver performance. Accid Anal Prev..

[3] Ward AF, Duke K, Gneezy A, Bos MW (2017). Brain Drain: The Mere Presence of One’s Own Smartphone Reduces Available Cognitive Capacity. J Assoc Consum Res..

[4] Yang C, Heinze J, Helmert J, Weitz J, Reissfelder C, Mees ST (2017). Impaired laparoscopic performance of novice surgeons due to phone call distraction: a single-centre, prospective study. Surg Endosc..

[5] Nikendei C, Jünger J. OSCE-Praktische Tipps Zur Implementierung Einer Klinisch-Praktischen Prüfung OSCE-Hands on Instructions for the Implementation of an Objective Structured Clinical Examination. . http://www.egms.de/en/journals/zma/2006-23/zma000266.shtml

[6] Majumder MAA, Kumar A, Krishnamurthy K, Ojeh N, Adams OP, Sa B (2019). An evaluative study of objective structured clinical examination (OSCE): students and examiners perspectives. Adv Med Educ Pract..

[7] Rivera-Rodriguez AJ, Karsh BT (2010). Interruptions and distractions in healthcare: Review and reappraisal. Qual Saf Heal Care..

[8] Balint BJ, Steenburg SD, Lin H, Shen C, Steele JL, Gunderman RB (2014). Do telephone call interruptions havean impact on radiology resident diagnostic accuracy?. Acad Radiol..

[9] Mentis HM, Chellali A, Manser K, Cao CGL, Schwaitzberg SD (2016). A systematic review of the effect of distraction on surgeon performance: directions for operating room policy and surgical training. Surg Endosc..

[10] Ghazanfar MA, Cook M, Tang B, Tait I, Alijani A (2015). The effect of divided attention on novices and experts in laparoscopic task performance. Surg Endosc..

[11] Weigl M, Stefan P, Abhari K (2016). Intra-operative disruptions, surgeon’s mental workload, and technical performance in a full-scale simulated procedure. Surg Endosc..

[12] Persoon MC, Van Putten K, Muijtjens AMM, Witjes JA, Hendrikx AJM, Scherpbier AJJM (2011). Effect of distraction on the performance of endourological tasks: A randomized controlled trial. BJU Int..

[13] Gillie T, Broadbent D. What Makes Interruptions Disruptive? A Study of Length, Similarity, and Complexity. Vol 50.; 1989.

[14] Srisarajivakul N, Lucero C, Wang XJ (2017). Disruptive behavior in the workplace: Challenges for gastroenterology fellows. World J Gastroenterol..

[15] Harvey R, Jarrett PG, Peltekian KM (1994). Patterns of paging medical interns during night calls at two teaching hospitals. CMAJ..

